# The Self‐Testing AfRica (STAR) Initiative: accelerating global access and scale‐up of HIV self‐testing

**DOI:** 10.1002/jia2.25249

**Published:** 2019-03-25

**Authors:** Heather Ingold, Ombeni Mwerinde, Anna Laura Ross, Ross Leach, Elizabeth L Corbett, Karin Hatzold, Cheryl C Johnson, Getrude Ncube, Rose Nyirenda, Rachel C Baggaley

**Affiliations:** ^1^ Unitaid Geneva Switzerland; ^2^ Malawi‐Liverpool‐Wellcome Trust Clinical Research Programme Blantyre Malawi; ^3^ Faculty of Infectious and Tropical Diseases London School of Hygiene and Tropical Medicine London UK; ^4^ Population Services International Washington DC USA; ^5^ Department of HIV/AIDS World Health Organization Geneva Switzerland; ^6^ Department of Infectious and Tropical Diseases London School of Hygiene and Tropical Medicine London UK; ^7^ Zimbabwe Ministry of Health Harare Zimbabwe; ^8^ Malawi Ministry of Health Lilongwe Malawi

**Keywords:** HIV testing, HIV self‐testing, market shaping, scale‐up, prevention, linkage to care, cost effectiveness

## Abstract

**Introduction:**

HIV self‐testing (HIVST) was first proposed as an additional option to standard HIV testing services in the 1980s. By 2015, two years after the first HIVST kit was approved for the American market and the year in which Unitaid invested in the “HIV Self‐Testing AfRica (STAR) Initiative,” HIVST remained unexplored with negligible access in low‐ and middle‐income countries (LMIC). However, rapid progress had been made. This commentary outlines the interlinked market, regulatory and policy barriers that had inhibited product development and kept HIVST out of LMIC policy. We detail the components of STAR that enabled rapid HIVST scale‐up, including critical investments in implementation, research, market forecasting, and engagement with manufacturers and regulators.

**Discussion:**

The STAR Initiative has generated crucial information about how to distribute HIVST products effectively, ethically and efficiently. Service delivery models range from clinic‐based distribution to workplace and partner‐delivered approaches to reach first‐time male testers, to community outreach to sex workers and general population “hotspots.” These data directly informed supportive policy, notably the 2016 WHO guidelines strongly recommending HIVST as an additional testing approach, and regulatory change through support for WHO prequalification of the first HIVST kit in 2017. In July 2015, only two countries had national HIVST policies and were implementing HIVST. Three years later, 59 countries have policies, actively implemented in 28, with an additional 53 countries reporting policies under development. By end‐November 2018 several quality‐assured HIVST products had been registered, including two WHO prequalified tests. STAR Initiative countries have drafted regulations governing *in vitro* diagnostics, including HIVST products. With enabling policies, pre‐qualification and regulations in place, donor procurement of kits has increased rapidly, to a forecasted estimate of 16 million HIVST kits procured by 2020.

**Conclusions:**

The STAR Initiative provided a strong foundation to introduce HIVST in LMICs and allow for rapid scale‐up based on the wealth of multi‐country evidence gathered. Together with sustained coordination and acceleration of market development work, HIVST can help address the testing gap and provide a focused and cost‐effective means to expand access to treatment and prevention services.

## 1 Introduction

HIV testing is the gateway to treatment and care and expanded prevention coverage. The first of the United Nations’ 90‐90‐90 Fast Track targets to end the HIV epidemic calls for 90% of people living with HIV (PLHIV) to know their HIV status by 2020 1. Access to and uptake of HIV testing services (HTS) in low‐ and middle‐income countries (LMICs) has increased substantially over the last three decades due to advances in treatment, rapid testing and greater availability of HIV testing in facility and community. Despite these advances, an estimated 25% of PLHIV globally still do not know their status 2. In eastern and southern Africa where the HIV burden is greatest, it is estimated that 2.7 million PLHIV still do not know their status 3, 4.

To address these gaps, innovative and strategic approaches to HIV testing are needed 5. HIV self‐testing (HIVST), has been highlighted as an additional tool to increase access and uptake of HIV testing in higher risk populations with low coverage and particularly in environments with high rates of stigma 6. In addition, HIVST has the potential to improve efficiency of the health system by triaging those without HIV straight to prevention services and freeing up health workers’ time and could consequently reduce costs of HTS 6.

HIVST was first proposed as an additional option to standard HTS in the 1980s 7. By 2015, three years after the first HIVST kit was approved for the American market 8, HIVST remained unexplored with negligible access in LMIC. By the end 2015, only two high‐income countries were actively implementing HIVST services as part of their public health HIV response primarily in the private sector, and WHO had yet to state an official position.

In 2013, WHO convened the first global consultation on HIVST identified that development of the necessary normative guidance for HIVST was largely hampered by lack of evidence on safety, acceptability, feasibility and scalability; uncertain distribution methods for HIVST kits; unclear processes for linking self‐testers to care and treatment; and lack of clarity on methods for creating demand among target populations 9, 10.

As with any new health technology, the introduction of HIVST kits in LMICs faced several immediate policy, regulatory and market challenges 11.

First, because of the lack of WHO prequalified products for self‐test use, opportunities to generate evidence for the public health benefit and to create demand were limited. Although the first discussion of HIVST introduction dates back to 1986, the first HIVST product only became available in 2012 when OraQuick^®^ In‐Home HIV Test (OraSure Technologies, Bethlehem, Pennsylvania USA) was approved by the US Food and Drug Administration 8, 12. It was not until mid to late‐2015 that Conformité Européenne‐marked products autotest VIH^®^ (AAZ Labs, Boulogne‐Billancourt France) and BioSURE HIV Self Test (BioSURE UK Ltd., Essex, England UK) became available for sale and use in the private sector in the United Kingdom and France.

Before 2015, there were no HIVST products registered by national regulatory authorities in Africa 11, 13, 14. At that time, while HIVST was being assessed through controlled research studies in the region, the market for, and awareness of, official HIVST in Africa, and LMICs more broadly, was extremely limited. Without evidence, WHO normative guidelines could not be made to support HIVST introduction – nor guidance on how it should be implemented, limiting countries’ ability to take on or prioritize HIVST as part of a national strategy. Furthermore, because of global and national level policy barriers, there were no regulatory pathways nor clear data collection systems to assure the quality of the HIVST products or to monitor the most ethical, acceptable and effective ways to implement self‐testing.

Second, a healthy HIVST market also requires solidified demand from end‐users and buyers including both national governments and donors. Before 2015, investment from governments and large‐scale donors was constrained by a lack of evidence that HIVST could be a safe and effective way to increase testing rates. Because of this, the President's Emergency Plan for AIDS Relief (PEPFAR) and the Global Fund to Fight AIDS, Tuberculosis and Malaria (Global Fund), two of the biggest procures of HIV test kits globally, had yet to procure HIVST outside of small quantities for research. At the same time, little was understood about potential levels of consumer demand for HIVST, including how consumers would want to access HIVST and the HIVST product attributes likely to drive uptake or impact outcomes.

Third, in the absence of HIVST guidelines and clearly defined quality assurance pathways, governments and donors had little ability to understand the degree by which various products met minimum quality standards. Without evidence to support paying a higher cost for self‐tests as opposed to professional use products, governments and donors signalled a very low willingness to pay. As a result, public sector procurement was largely frozen. A competitive market with several manufacturers has driven down the cost of professional‐use HIV rapid diagnostic tests. While these issues apply to HIVST kits, they also face further challenges as they must be designed and packaged for self‐test use, which incurs additional costs, and requires additional approvals and regulations. Faced with the prospect of low margins, unclear approval pathways, and limited concrete volumes, manufacturers responded by minimizing investment and adapting professional use products for self‐testing. In addition, several suppliers with promising products lacked deep experience operating in LMIC markets 15.

Addressing the interlinked market, regulatory and policy barriers that had inhibited HIVST product development through a comprehensive approach was the goal of the Unitaid investment through the HIV Self‐Testing AfRica (STAR) Initiative.

This commentary provides a broad overview of the strategies and achievements of the STAR Initiative in the introduction of HIVST in LMICs and the development of the HIVST market and considers the remaining challenges for bringing HIVST to scale.

## 2 Discussion

In 2015 Unitaid invested in a comprehensive effort to develop the market for HIVST by:

1. Establishing the evidence for its safety, acceptability, feasibility and scalability;
2. Creating an enabling environment with regards to normative guidelines, national policies, and regulatory frameworks based on the foundation of research evidence;
3. Generating diverse demand through multiple distribution channels adapted to the needs of priority populations and create advocacy for additional financing; and
4. Accelerating market entry for suppliers at affordable and sustainable prices.

**Figure 1:**
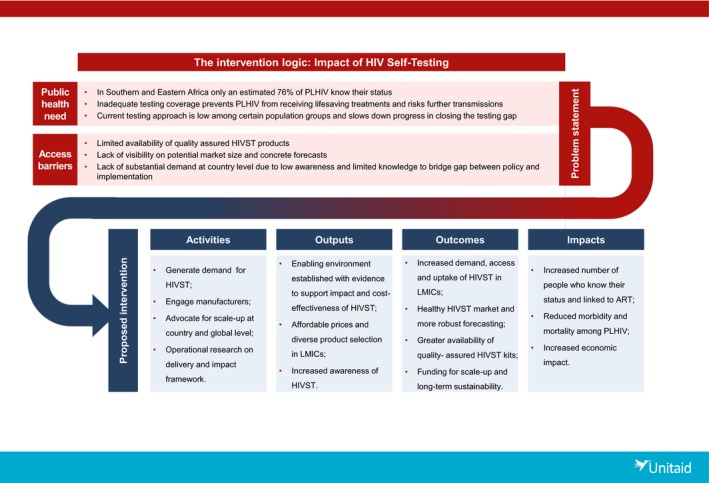
Unitaid‐PSI HIV STAR Project: implementation for impact PSI, Population Services International; STAR, Self‐Testing AfRica; PLHIV, people living with HIV; HIVST, HIV self‐testing; LMIC, low‐ and middle‐income countries.

This commitment resulted in the support of the five‐year “HIV Self‐Testing in AfRica (STAR) Initiative” with Population Services International (PSI) and a consortium of partners, including London School of Hygiene and Tropical Medicine, Liverpool School of Tropical Medicine, University College London, and Society for Family Health (SFH) and the University of Witwatersrand Reproductive Health and HIV Institute (Wits RHI) in South Africa, initially in three Southern African countries (Malawi, Zambia and Zimbabwe), expanding to include South Africa, Lesotho and Swaziland in 2017 (Figure 1).

### 2.1 Evidence framework for safety, acceptability, feasibility and scalability

An increasingly strong set of evidence from a range of different populations and settings, demonstrates that the implementation of HIVST is safe, acceptable and effective when kits are used correctly, and can contribute to increased HIV testing coverage 6, 16, 17.

The STAR Initiative partners worked collaboratively to identify, design and implement a research agenda to inform normative guidelines, the foundation upon which many national health policies are based. This was a major turning point for the creation of an enabling environment since most countries would not adopt policies for HIVST and donors would not invest in the product without these guidelines.

The agenda, which was in part funded by other partners, included formative research and accuracy studies to establish that HIVST was acceptable, safe, and could be correctly performed by priority populations 18, 19. Further studies tested user preferences and established simple distribution models aimed at using HIVST for maximum public health impact 18, 20, 21. Data from these early distribution models informed costing and cost‐effectiveness studies to establish the evidence for HIVST scalability and sustainability 20, 22, 23. This collaboration helped generate important aspects of the evidence needed to inform WHO Guidelines on HIVST and Partner Notification (i.e., HIVST normative guidelines) published in December 2016, and the operational guidance at country level that followed.

### 2.2 Creating an enabling environment for national policies, and regulatory frameworks

The STAR Initiative supported efforts to widely disseminate the guidelines and contribute to national policy and strategy development, informing HIVST roll‐out across LMICs. As policy was being established, the STAR Initiative worked with country governments in Southern Africa through technical working groups (TWGs) to ensure that research was continuously informing implementation. The TWGs were chaired by MoHs and included representatives from the national regulatory bodies, WHO, in‐country donors supporting national HIV programmes, civil society and PLHIV advocacy and support groups, as well as PSI and local research partners. To increase knowledge‐sharing across countries and stakeholders, and to drive development of an enabling environment beyond STAR countries, the STAR Initiative developed toolkits to guide research design and HIVST implementation and met on a regular basis to share experiences. This included direct country exchanges between STAR Initiative countries and other African countries.

The STAR Initiative hosted a pivotal workshop in Nairobi in April 2017, linked to a WHO regional guidelines workshop, to share their HIVST implementation experience with MoH representatives. This workshop provided the information necessary to transform normative guidance into implementation and led to a series of country implementation visits, catalysing action across Africa such as MoH tours of STAR implementation projects to learn directly about challenges and successes (e.g. eSwatini, Lesotho, Malawi, Uganda, and Zimbabwe). Botswana and Mozambique HIVST policy adoption and/or pilots were initiated as a result of this meeting. A number of West African countries (e.g. Côte d'Ivoire, Mali, and Senegal) were approved for funding by Unitaid through the ATLAS project to pilot HIVST shortly after the workshop. Another workshop was organised in Bangkok in collaboration with Unitaid, WHO and UNAIDS in October 2018, to share evidence and experience with HIVST with governments and implementers from 13 countries in Asia and the Pacific including the launch of the WHO strategic framework on HIVST 24 to accelerate the introduction and scale‐up of HIVST in this region [Meeting report forthcoming].

To support the operationalization of policy, the STAR Initiative provided technical assistance and held regional workshops to clarify diagnostic regulatory frameworks and identify the steps necessary to establish external quality assurance and post‐market surveillance systems for HIVST 25. As a result, targeted solutions were developed in each country and included activities such as establishing medical device committees, defining responsibilities of regulatory bodies, introducing laws to parliament for product oversight, and evaluating and registering products. Harmonization of these regulatory processes has been desired since the first diagnostic products were introduced in Africa.

As a result of these efforts and advocacy, momentum around HIVST is increasing and many additional countries have indicated their interest in introducing HIVST. As of July 2018, 59 countries now have policies explicitly allowing HIVST, of which 28 are now fully implementing 26. In addition, 32 countries are actively piloting HIVST 26.

### 2.3 Generating diverse demand through multiple distribution channels and advocacy for additional financing

For a developed and sustainable market, demand for HIVST products must be generated from end‐consumers, MoHs and donors. The STAR Initiative conducted in‐depth research to understand consumer preferences and provider perceptions of HIVST and used this evidence to inform and refine the design of eight facility‐ and community‐based distribution models in Malawi, Zambia and Zimbabwe 18, 20. In its first two years, the STAR project showed that community‐based and partner‐delivered HIVST can be an effective testing approach with several advantages that complement conventional options 26, 27. HIVST also demonstrated the ability to reach those who are not currently accessing services, such as first‐time testers, and facilitated frequent re‐testing, particularly among those with high ongoing risk 28. HIVST may also improve efficiency and effectiveness of overburdened health systems, by refocusing testing services and resources on those with a reactive self‐test result in need of confirmatory testing, thereby increasing the efficiency of conventional testing systems 29.

A concern about HIVST is the need to maximize rapid links to confirmatory testing and antiretroviral therapy (ART) for people with reactive tests, in addition to linking those with a non‐reactive test to appropriate and effective prevention services, such as pre‐exposure prophylaxis (PrEP) and voluntary medical male circumcision (VMMC). Research and programmatic data from STAR has demonstrated that HIVST offered by community mobilizers can increase demand, motivation for and uptake of VMMC 27. In a community‐based HIVST project in Zimbabwe, demand for ART was significantly increased, with survey data also showing that linkage to confirmatory testing and ART initiation among those who tested positive and were not previously on ART ranged between 30% and 46% six weeks after an HIVST campaign 30. Assuming the same level of ART initiation among those newly diagnosed, HIVST is likely to result in additional 60,000 to 230,000 PLHIV receiving life‐saving treatment annually from 2020 or combined total of 110,000 to 460,000 (2019 to 2020), in addition to significant linkage to appropriate prevention options, including PrEP 28.

A review of evidence in sub‐Saharan Africa, including evidence from the STAR Initiative, indicates that delivery models have had varying acceptability across different targets 29. Additional evidence shows that the use of HIVST can be cost‐effective 31. However, to maximize population health impact within the budget available, HIVST needs to be targeted based on the prevalence of undiagnosed HIV, likely HIV incidence, and the overall costs of delivering this testing modality. For example, with community‐based HIVST likely to be cost‐effective if introduced for women having transactional sex and adult men, provided that the undiagnosed HIV prevalence is above 3% and when delivered through campaign distribution, such as every five years 31.

Consistent engagement with MoH, Global Fund and PEPFAR through the routine dissemination of the STAR Initiative's findings allowed these key partners to integrate HIVST into longer‐term scale‐up plans and funding, including within Global Fund concept notes and PEPFAR country operational plans. In 2018, HIVST was included as a dedicated testing strategy in PEPFAR country guidance and received a substantial funding increase. Efforts are also underway to embed HIVST into domestic health budgets and determine the most cost‐effective way of delivering testing services, including HIVST. For example, to enable the transition to domestic financing in South Africa, the potential cost‐savings of HIVST will be quantified and incorporated into the HIVST investment case being developed for the South African National Department of Health.

### 2.4 Accelerating market entry for suppliers at affordable and sustainable prices

Without normative guidance, demand for HIVST kits was weak, with uncertain forecasts ranging from 4 to 80 million tests until 2016. To provide greater clarity on potential demand, with funding from the Bill and Melinda Gates Foundation (BMGF), PSI developed estimates of the size of the HIVST market. PSI estimated that by 2020, the market would reach either 3.3 to 5.7 million or 11 to 15 million tests per year in nine African countries depending upon whether conservative or moderate assumptions were applied 15.

Based on the “moderate scenario” of the Expanding Access to HIVST report, which assumes that donors will invest to support development of both public and private sector markets, the HIVST market size could be between 13 and 15 million kits annually by 2020 26. Currently 99 countries had included HIVST in procurement planning, representing more than 85% of the global HIV burden. Preliminary estimates based on these findings suggest HIVST is likely to result in at least 200,000 to 500,000 additional PLHIV who will know their status annually.

In order to ensure a high‐quality supply of HIVST kits, regulators and manufacturers needed to understand how HIVST fits into the current regulatory structures. Under the STAR Initiative, WHO worked to clarify the pre‐qualification (PQ) requirements with manufacturers. By leveraging the accuracy and usability studies conducted in‐country, PSI was able to identify product and instruction changes needed to achieve PQ or Global Fund/Unitaid Expert Review Panel for Diagnostics (ERPD) approval and national registration for OraQuick^®^, the only product with the evidence required to submit a dossier at the time. Wits RHI simultaneously conducted studies in South Africa to inform the WHO PQ submissions for several HIVST products, including blood‐based products. However, as of 2016, significant gaps remained between the available evidence for blood‐based HIVST versus oral‐fluid HIVST. This gap had the potential to hinder diverse market supply. With the support of BMGF, the STAR Initiative conducted consumer usability studies on four blood‐based HIVST products, optimizing instructions for use, and piloting the use of the most suitable blood‐based HIVST products 32.

To improve the availability of information about the HIVST market, Unitaid and WHO developed a series of HIVST landscape reports to inform demand, assist with product selection and application, and incentivize supplier participation 26, 33. The landscape reports included a summary of the key evidence to support the use of HIVST, the current state of HIVST policy and regulation, and detailed information about available and pipeline HIVST products. The landscape reports also included information on HIVST demand.

Despite this progress towards international approvals, manufacturers remain concerned that in some countries the regulatory process remains opaque, the responsible authorities for the registration of HIVST products are still unclear, and even with WHO PQ, in‐country validation and registration are still required. These complications add to the cost of doing business for manufacturers and threaten the sustainability of affordable prices for HIVST.

## 3 Remaining market challenges

Despite this growing interest, however, the HIVST market is still nascent and the current volumes are unlikely to be sufficient to make the LMIC market healthy and attractive to multiple suppliers. Market conditions are further compounded by continued ambiguity surrounding forecasting and regulatory environments. High volumes of demand for HIVST kits, and HIV testing in general, will be needed to establish the viable market necessary for long term sustainability of HIVST. Approximately 2.5 million HIVST kits were sold worldwide between 2012 and 2017 33. In July 2018, the first global HIVST forecast showed at least 5 million HIVST kits would be procured by the end of 2018 and that with current donor and private sector investments the market would reach nearly 20 million kits by 2020 26.

Currently there are only two WHO prequalified HIVST products, the INSTI HIV Self Test (bioLytical, Richmond, British Columbia Canada) and the OraQuick HIV Self‐Test (OraSure Technologies), with others under review 34. Through the Global Fund/Unitaid ERPD four other HIVST products, all of which are blood‐based, have also become available for use in the context of operational research and demonstration projects 35.

While HIVST has been shown to be effective, the investment in additional testing is being scrutinized. In countries where the first 90‐90‐90 target has been reached or nearly attained, diagnosing the few people with HIV who do not know their status may be challenging and costly. In countries with slower progress towards achieving the first 90 target, a major shift will be needed in the approach to testing to improve effectiveness and efficiency in finding those with an undiagnosed HIV infection. There is currently still lacking clarity on how HIVST can fit and contribute to reaching the remaining PLHIV in need of diagnosis and contributing to uptake of prevention. Creating an investment case that is country context specific is critical to the sustainability and accelerated scale up of HIVST.

Notwithstanding these challenges, the prospects for the HIVST market in LMICs have improved notably over the last few years. This is due in no small measure to significant investments committed by Unitaid and other partners, including the BMGF and the Children's Investment Fund Foundation's Charitable Support Agreement with OraSure (2017) which reduced the price of the only WHO Prequalified product to US$2 for 50 countries 36. This agreement is only valid for four years, and the price may increase if the requisite volumes are not established and maintained by the end of the term. An increase in demand and adoption as well as the presence of other affordable products will reduce this threat and offer countries a choice in product selection based on the preferences of different user populations.

Despite having a better understanding of how to deliver HIVST and an increasing number of countries introducing it, coverage remains low in comparison to the current need, and this is due to limited awareness of users and providers. Both the demand and supply for HIVST remain limited, hindering the establishment of a healthy market with affordable products.

While the findings from the STAR Initiative have been successful in motivating country governments and funding agencies, the need for additional evidence of cost‐effectiveness of the different HIVST delivery models is critical to securing increased scale‐up funding. Likewise, there is the need to demonstrate the impact of HIVST in enabling countries to meet their HIV coverage targets as part of the UN 90‐90‐90 to make a compelling investment case. Increased effectiveness of HIVST will require a balance between targeting delivery for high testing coverage for new testers versus benefits from re‐testers. Such a balance is important to achieve efficiencies in the health system because health services and resources will be focused on confirmatory testing and linkage to treatment while those with a negative test can be triaged to prevention services 6.

## 4 Conclusions

The STAR Initiative has provided a strong foundation to introduce HIVST, in LMICs, and allow for rapid scale‐up based on collection of multi‐country evidence and rapid dissemination to inform policy and practice through national TWGs, international workshops, and regulators and manufacturers fora. The increasingly strong evidence base has shown that HIVST is preferred by many Africans to all other testing modalities and can reach those who do not test and are at high risk of HIV in LMICs. The market for HIVST is expanding and national HIV programmes, with support from external donors, are beginning to move beyond the policy and pilot stage to scale up HIVST through multiple distribution models. Ensuring an enabling environment with systems and structures in place that are supportive of HIVST, having safeguards in place to keep inferior products out of LMIC markets, to prevent social harms, and effective targeting of low cost models for HIVST distribution and linkage into prevention and care services in high prevalence populations will maximize the important potential for public health impact. Together with sustained coordination and acceleration of market development work outside of the Africa region, HIVST can help address the testing gap and provide a focused and cost‐effective means to expand access to treatment and prevention services.

## Competing interests

The authors declare that they have no competing interests.

## Authors’ contributions

H.I., O.M. and A.L.R. made substantial contributions to formalize the concept of the commentary and development of the draft. R.L. contributed to the concept and evolution of the commentary. R.B., E.C., K.H. and C.J. have been involved in reviewing the commentary and providing valuable input. All authors have given final approval of the version to be published.
